# TIARP attenuates autoantibody-mediated arthritis via the suppression of neutrophil migration by reducing CXCL2/CXCR2 and IL-6 expression

**DOI:** 10.1038/srep38684

**Published:** 2016-12-20

**Authors:** Asuka Inoue, Isao Matsumoto, Yuki Tanaka, Naoto Umeda, Chinatsu Takai, Hoshimi Kawaguchi, Hiroshi Ebe, Hiroto Yoshida, Yoshihiro Matsumoto, Seiji Segawa, Satoru Takahashi, Takayuki Sumida

**Affiliations:** 1 Division of Rheumatology, Department of Internal Medicine, Faculty of Medicine, University of Tsukuba, 1-1-1 Tennodai, Tsukuba 305-8575, Japan; 2 Chugai Pharmaceuticals Co., Ltd. Fuji Gotemba Research Labs, 1-135 Komakado, Gotemba, Shizuoka 412-8513, Japan; 3 Department of Anatomy and Embryology, Doctoral Program in Biomedical Sciences, Graduate School of Comprehensive Human Sciences, University of Tsukuba, Tsukuba, 305-8575, Japan

## Abstract

TNFα-induced adipose-related protein (TIARP) is a six-transmembrane protein expressed on macrophages, neutrophils and synoviocytes. We reported recently that mice deficient in TIARP (TIARP^−/−^) spontaneously develop arthritis and are highly susceptible to collagen-induced arthritis (CIA) with enhanced interleukin (IL)-6 production. However, the effects of TIARP on neutrophils and fibroblast-like synoviocytes (FLS) have not been elucidated. We analyzed the roles of TIARP in K/BxN serum transfer model using TIARP^−/−^ mice. Arthritis in TIARP^−/−^ mice transferred with K/BxN serum was significantly exacerbated compared with WT mice. We characterized the differences in neutrophils between wild-type (WT) and TIARP^−/−^ mice by DNA microarray. Overexpression of CXCR1 and CXCR2 was noted in TIARP^−/−^ neutrophils. Neutrophils of TIARP^−/−^ mice showed strong migration activity, which was markedly facilitated by CXCL2 *in vitro* and *in vivo*. Moreover, enhanced production of CXCL2 and IL-6 and cell proliferation was noted in TIARP^−/−^ TNFα-stimulated FLS. Blockade of IL-6R significantly attenuated serum-transferred TIARP^−/−^ arthritis with diminished neutrophil recruitment in joints. Our findings suggested that TIARP independently down-regulated CXCL2 and IL-6 production by FLS, and the expression of chemokine receptors (CXCR1 and CXCR2) in neutrophils, with resultant reduction of neutrophil migration into arthritic joints.

Rheumatoid arthritis (RA) is a systemic inflammatory disease characterized pathologically by hyperplasia of synovial tissues and destruction of cartilage and bone^1^. Accumulation of inflammatory cells in the synovial fluid (SF), especially neutrophils, is thought to play an important role in the pathogenesis of RA^2^,^3^,^4^.

We have reported previously the upregulation of TNFα-induced adipose-related protein (TIARP) [also known as six-transmembrane protein of prostate 2 (STAMP2) and tumor necrosis factor α-induced protein 9 (TNFAIP9)] in glucose-6-phosphate isomerase (GPI)-induced arthritis, especially in CD11b^+^ splenocytes and synovia of mice^5^. TNFα-induced up-regulation of TIARP expression is also noted in adipocytes during adipose differentiation^6^. Apart from TNFα, TIARP expression is also up-regulated by other inflammatory cytokines and mediators, such as interleukin (IL)-6, IL-1β, lipopolysaccharide (LPS) in various cells (e.g., macrophages, hepatocytes)^6^,^7^,^8^,^9^,^10^. On the other hand, downregulation of TIARP has been described in diabetes mellitus, atherosclerosis and arthritis^11^,^12^,^13^. In this regard, we reported previously that TIARP^−/−^ mice spontaneously develop polyarthritis and are susceptible to collagen-induced arthritis (CIA)^13^. Moreover, high expression levels of pro-inflammatory mediators (e.g., TNFα, IL-6 and CXCL2) and marked accumulation of neutrophils and macrophages have been described in the arthritic joints of TIARP^−/−^ mice^13^.

In macrophages, TIARP can suppress the production of pro-inflammatory cytokines by inhibiting nuclear factor-kappa B (NF-κB) and signal transducer and activator of transcription 3 (STAT3) signaling pathways^13^. Human TIARP counterparts, such as six transmembrane epithelial antigen of prostate 4 (STEAP4), are also highly expressed in peripheral blood mononuclear cells (PBMC) and synovial CD68^+^ cells in patients with RA^14^,^15^. Furthermore, STEAP4 mRNA expression level correlates with the number of neutrophils in the peripheral blood of RA patients^16^. However, the role of TIARP/STEAP4 expressing cells other than macrophages, such as neutrophils and fibroblast synoviocytes (FLS) in inflammatory arthritis is poorly understood.

The K/BxN serum transfer model has greatly facilitated our understanding of the role of effector cells and molecules in the induction of arthritis. K/BxN serum contains pathogenic autoantibodies against glucose-6-phosphate isomerase (GPI), which form immune complexes (ICs) on the cartilage surface^17^,^18^,^19^,^20^. In addition to these antibodies, neutrophils, mast cells, and macrophages are also considered to play important roles in the pathogenesis of arthritis in this model^3^,^21^,^22^. Thus, in both human RA and mice models, neutrophils are recruited into the affected joints by chemoattractants where they play a role in worsening tissue damage^2^,^5^,^23^. In addition, IL-1 and TNFα, but not IL-6, are involved in the development of serum-related arthritis^17^,^24^.

The purpose of the resent study was to further define the role of TIARP in arthritis. The results showed that downregulation of TIARP was associated with exacerbation of K/BxN arthritis, and that this effect was mediated through massive intra-articular migration of neutrophils. The latter was mainly mediated through upregulation of CXCR1/2 expression on neutrophils and overproduction of CXCL2 and IL-6 from FLS. Taken together with our previous work^13^, this study provides new evidence that TIARP plays a key role as a negative regulator by suppressing not only activated macrophages, but also cross-talk between infiltrating neutrophils and proliferated FLS via CXCL2/CXCR2 and IL-6, in the pathogenesis of arthritis.

## Methods

### Mice

TIARP^−/−^ mice were generated as described previously^13^. KRN-transgenic mice were kindly provided by Drs. D. Mathis and C. Benoist (Harvard Medical School, Boston, MA) and Dr. Koichiro Ohmura (University of Kyoto, Kyoto, JAPAN). K/BxN mice were generated by crossing KRN-transgenic mice with NOD mice. All mice were kept under specific pathogen-free conditions in an environmentally controlled clean room at the University of Tsukuba. All animal experiments were approved by the institutional animal care committee of the University of Tsukuba, and conducted in accordance with the institutional ethics guidelines of the University of Tsukuba.

### K/BxN serum transfer arthritis

Serum samples were collected from 6- to 8-week-old K/BxN mice and pooled for each experiment. Arthritis was induced by intraperitoneal injection of 50 μl of K/BxN mouse serum on days 0 and 2. The thickness of each ankle joint was measured with a caliper.

### Histopathology

The ankle joints were dissected out and fixed tissues with neutralized 10% formalin. The tissues were decalcified in 10% formic acid and embedded in paraffin. Serial sections (4-mm thick) were stained with hematoxylin-eosin (H&E) and assessed histologically as described previously^13^.

### Flow cytometric analysis

For flow cytometry, cells were stained with FITC-, PE-, PerCP-, or APC-conjugated monoclonal antibodies (mAbs). Rat mAbs to mouse CD11b, CD45.1, CD45.2, CXCR2, F4/80 and Gr-1 were purchased from Biolegend (San Diego, CA). Cell surface was stained using standard techniques, and the cells were stained with a FACSVerse cytometer (Becton Dickinson, Franklin lakes, NJ) using Flowjo software (Tree Star, Ashland, OR).

### Depletion of neutrophils and monocytes/ macrophages

For depletion of neutrophils, mice were injected intravenously with 0.5 mg of control IgG or anti-Gr1 mAb (RB6-8C5) one day before the induction of arthritis every 3 days. For *in vivo* depletion of monocytes/macrophages, mice were injected intraperitoneally with 200 μl control liposomes or clodronate liposomes on days 1 and 6 after the induction of arthritis. Depletion of neutrophils and monocytes/macrophages was confirmed by fluorescence activated cell sorting (FACS) analysis in all experimental animals.

### Generation of bone marrow chimeras

Bone marrow cells were obtained from the femurs and tibias by flushing, erythrocytes were lysed with a lysis buffer. Bone marrow chimeras were established by instillation of 2 × 10^7^ bone marrow cells in 1 × Hanks’ balanced salt solution (HBSS) into 8-week-old lethally irradiated (single dose of 9 Gy) recipient mice via tail vein injection. The chimera mice were maintained for 4 weeks after bone marrow transfer. Mixed bone marrow chimera were generated by reconstituting the recipient’s bone marrow with bone marrow from two different donor strains at a 1:1 ratio.

### Microarray analysis

Total RNA from TIARP^−/−^ or WT neutrophils was isolated using an RNeasy Mini Kit (Qiagen, Hilden, Germany). The relative purity of the RNA was measured using an Agilent 2100 Bioanalyzer. Total RNA was amplified and labeled. Agilent Feature Extraction version 10.7.3.1 image analysis software was used to extract data from raw microarray image files. The mRNA expression profile has been uploaded to GEO database (#GSE73306), and then subjected to normalization and log transform treatment. The differentially expressed genes (DEGs) were obtained by folds change analysis. Functional Annotation Chart in DAVID (http://david.abcc.ncifcrf.gov/) is able to identify the most relevant biological terms associated with a given gene list.

### Quantitative real-time PCR

Total RNA was isolated by the ISOGEN (Nippon gene, Tokyo) extraction method according to the instructions provided by the manufacturer. Quantitative real-time PCR was performed as described previously^13^ using the TaqMan gene expression assay (Applied Biosystems, Foster City, CA). Real-time PCR was carried out using the ABI7500 (Applied Biosystems). Analysis of post-PCR melting curves confirmed the specificity of the single target amplification. The expression of each gene was determined relative to that of *Gapdh*.

### Isolation of pro-inflammatory cells and FLS from ankle joints

The skin was removed from arthritic hind paws, and synovial cells were harvested and then digested with 1.6 U/ml Liberase^TM^ (Roche) for 60 min at 37 °C. After enzymatic digestion, the number of pro-inflammatory cells in the ankle joint was analyzed by flow cytometry, and synovial cells were pelleted by centrifugation, plated in Dulbecco’s modified Eagle medium (DMEM) supplemented with 10% fetal calf serum (FCS), penicillin, streptomycin, and L-glutamine, and maintained in a humidified atmosphere of 5% CO_2_ at 37 °C. After overnight incubation, nonadherent cells were removed and adherent cells were cultivated under the conditions described above. Upon reaching confluence, they were detached with trypsin/ethylenediaminetetraacetic acid (EDTA), and recultured under the same conditions. All experiments were performed using FLS passages 3–6.

### Cell proliferation assay

The BrdU assay was used to examine the role of TIARP on proliferation of FLS. The test is based on measuring BrdU incorporation during DNA synthesis. FLS (1 × 10^5^/well) were cultured for 24 hours in 96-well plates in DMEM, either alone or in the presence of 100 ng/ml TNFα or 100 ng/ml LPS. The incorporation of BrdU into newly synthesized DNA was measured using an enzyme-linked immunosorbent assay (ELISA) kit (Roche Applied Science, Indianapolis, IN) and the protocol recommended by the manufacturer.

### Chemotaxis assay

Trans-well chemotaxis assay was performed in 24-well transwells (6.5-mm diameter, 3-μm pore size; Corning, New York, NY). Neutrophils were isolated from splenocytes using anti-mouse Ly6G MACS beads from Milenyi Biotec (Auburn, CA). Neutrophils (1 × 10^5^/ml) were added to the upper chamber, while RPMI-1640 medium (with or without 10 or 100 ng/ml recombinant mouse CXCL2) was added to the lower chamber. After 3 hours of incubation, neutrophils that had migrated into the lower chamber were collected and counted. Criss-cross coculture was prepared with neutrophils in the upper wells and supernatants from FLS-stimulated with or without TNFα in the lower wells. The supernatant was diluted in culture medium at 1:1 ratio and applied to the lower chamber. To verify the effect of CXCL2 on the chemotactic activity, we performed the chemotaxis assay, which involved adding 10 ng/ml of goat anti-CXCL2 antibody (R&D Systems, Minneapolis, MN) or control IgG to the lower chamber.

### Enzyme-linked immunosorbent assay

ELISA kits (R&D Systems) were used to measure cytokines and chemokines in cell culture supernatants and also the serum concentrations of TNFα, IL-6, IL-1β and CXCL2.

### Treatment of arthritis with anti-IL-6R Abs and TNFR-Fc

To neutralize proinflammatory cytokines, mice were injected intraperitoneally with 2 mg of anti-IL-6R Abs, or 1 mg of TNFR-Fc (etanercept), or control IgG (purified from the serum of non-immunized rats) every 2 days after induction of K/BxN serum-induced arthritis.

### Statistical analysis

Data were expressed as mean ± SEM. Differences between groups were evaluated for statistical significance by the Student’s *t*-test, while differences in the incidence of K/BxN serum-induced arthritis were evaluated by the χ^2^-test and in the severity score by the Mann-Whitney U-test. A two-tailed P value of <0.05 was considered significant.

## Results

### Exacerbation of serum-transferred arthritis in TIARP-deficient mice via marked intra-articular migration of neutrophils

**Figure 1:**
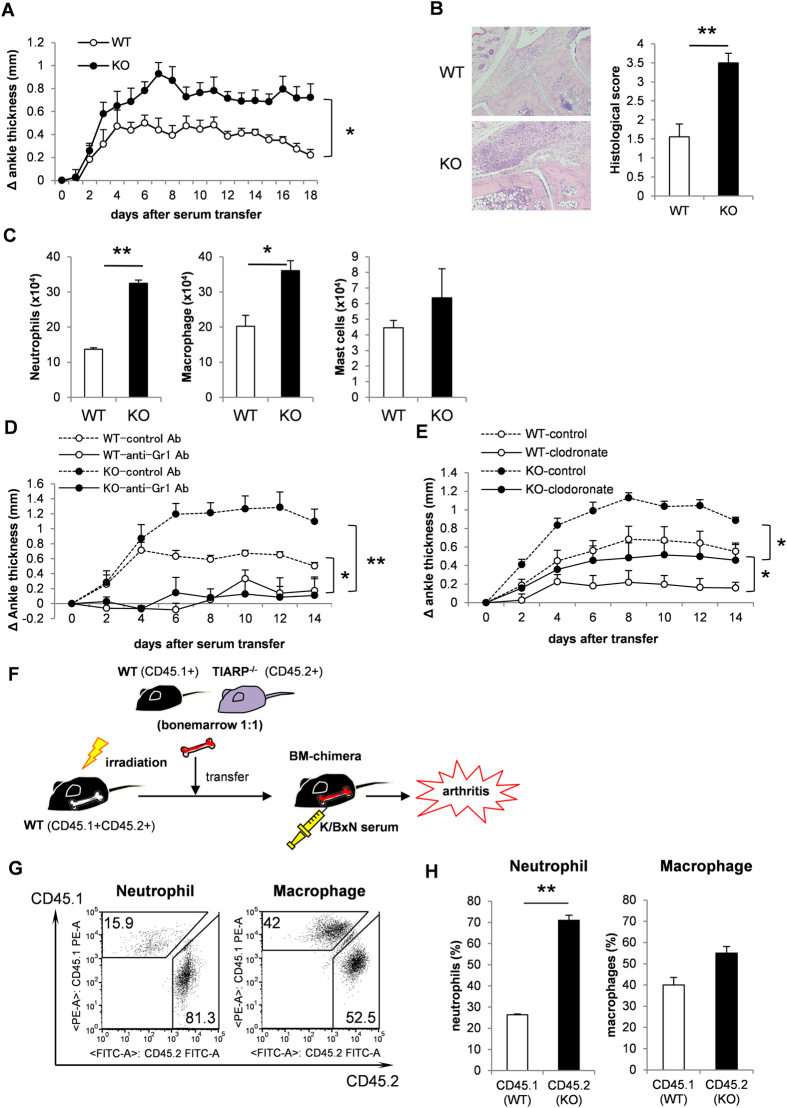
Development of K/BxN serum-induced arthritis in mice. For induction of arthritis, 50 μl of K/BxN serum was injected intraperitoneally on days 0 and 2. (**A**) Delta (⊗-) ankle thickness (mm). Data were obtained from two independent experiments involving WT mice and TIARP^−/−^ mice (n = 13, each). (**B**) Hematoxylin and eosin (H&E) stained sections of the ankle joints after induction of arthritis. Original magnification x100. Inflammation scores in WT and TIARP^−/−^ mice after induction of arthritis. (**C**) Numbers of neutrophils, macrophages and mast cells in the ankle joints determined on day 14. (**D**) Mice were administered arthritogenic K/BxN serum and either isotype control (rat IgG2b) or anti-Gr1 mAbs (RB6-8C5). The dose of mAbs was 200 μg per injection every 2 days throughout the experiment. (**E**) Changes in ankle thickness in clodronate liposome- and control liposome-treated WT and TIARP^−/−^ mice. Data are representative of two independent experiments. (**F**) Experimental scheme. Neutrophils from WT (CD45.1^+^) and TIARP^−/−^(CD45.2^+^) bone marrow were adoptively transferred with each cells intravenously into WT (CD45.1^+^ CD45.2^+^) mice, which was treated with K/BxN serum on day 2. (**G**,**H**) Frequency of migrated neutrophils and macrophages in the joint after 6hrs of serum transfer were detected by flow cytometry. Data are mean mean ± SEM. *P < 0.05, **P < 0.01.

To assess the effect of TIARP on the development of K/BxN serum-transferred arthritis, TIARP^−/−^ mice were injected with K/BxN serum using the standard protocol described above. The severity of arthritis was markedly worse in TIARP^−/−^ mice than WT mice (Fig. 1A). Consistent with these findings, histological examination of the ankle joints harvested at day 7 after serum transfer demonstrated significantly enhanced joint inflammation in TIARP^−/−^ mice (Fig. 1B). This was accompanied by significantly larger accumulation of neutrophils and macrophages, but not mast cells, into the ankle joints (Fig. 1C).

We have already confirmed the importance of macrophages in arthritis in TIARP^−/−^ mice^13^. To determine the role of neutrophils in the development of serum-transferred arthritis in TIARP^−/−^ mice, we first treated these mice with the neutrophil-specific depleting monoclonal antibody RB6-8C5 (Gr1). Anti-Gr1 mAb significantly inhibited the development of arthritis, compared with isotype control (Fig. 1D). In other experiments, clodronate liposomes (to reduce the number of macrophages/monocytes (Fig. 1E) selectively depleted macrophages/monocytes, but not neutrophils, in the spleen of both WT and TIARP^−/−^ mice (data not shown). Although clodronate liposome-treated WT mice showed milder severity of arthritis than control liposome-treated WT mice, clodronate liposomes-treated TIARP^−/−^ mice developed more severe arthritis relative to the that observed in the control liposome-treated WT mice.

To further determine whether TIARP^−/−^ neutrophils and macrophages were recruited into the site of inflammation *in vivo*, 1:1 mixture of WT (CD45.1^+^) and TIARP^−/−^ (CD45.2^+^) bone marrow cells were adoptively transferred into irradiated WT (CD45.1^+^ CD45.2^+^) mice immediately before K/BxN serum transfer (Fig. 1F). Such transfer induced significant recruitment of TIARP^−/−^ (CD45.2^+^) neutrophils, but not macrophages, in the ankle joint after the induction of arthritis, compared with WT (CD45.1^+^) mice (Fig. 1G,H). Collectively, these data demonstrate that TIARP deficiency results in increased recruitment of neutrophils with resultant exacerbation of K/BxN serum-induced arthritis.

### Up-regulation of chemokine receptors in TIARP^−/−^ neutrophils

Since neutrophils play an important role in inflammatory arthritis in TIARP^−/−^ mice, we used microarray analysis to investigate the molecular difference in neutrophils isolated from splenocytes (GEO accession number: GSE73306) between WT and TIARP^−/−^. Genes were differentially expressed into two main clusters in TIARP^−/−^ neutrophils; 606 up-regulated genes and 1,010 down-regulated genes, compared with WT neutrophils. To understand the functional categories of the up-regulated genes, we used DAVID GO enrichment analysis. Gene ontology (GO) analysis of the upregulated genes in TIARP^−/−^ neutrophils demonstrated high proportion of genes involved in chemotaxis and immune response (Table 1).

**Figure 2:**
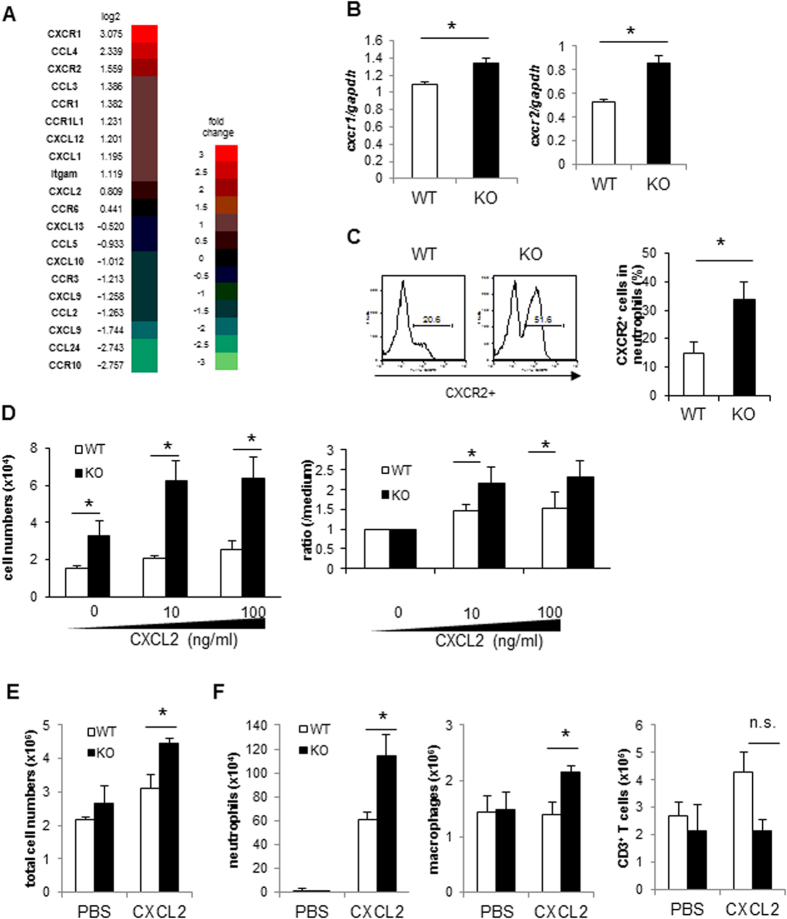
Enhanced expression of CXCR1/CXCR2 and migration of neutrophils. (**A**) Expression of genes related to chemotaxis as determined by microarray analysis. The mRNA level in TIARP^−/−^ neutrophils relative to that in WT neutrophils. Fold change >1 indicates enhanced gene in TIARP^−/−^ neutrophils compared to WT neutrophils. (**B**,**C**) Neutrophils from splenocytes of WT and TIARP^−/−^ mice. The expression levels of CXCR1 and CXCR2 were analyzed by qPCR (**B**) and by flow cytometry (**C**). (**D**) Using Boyden chambers, the isolated neutrophils were seeded in the upper wells, and recombinant mouse CXCL2 (10, 100 ng/ml) was added to the lower wells. After 3 hr-incubation, migrated neutrophils were collected and counted. (**E**,**F**) Mice were injected intraperitoneally with 0.5 μg of CXCL2. After 4 hr, total peritoneal exudate cells (PECs) were counted (**E**). Neutrophils, macrophages, and CD3+ T cells were counted at 4 hr after injection (**F**). Data are mean ± SEM. *P < 0.05.

Figure 2A summarizes the genes involved in chemotaxis in TIARP^−/−^ neutrophils. The observed patterns of gene expression were validated by quantitative PCR of neutrophils. Thus, upregulation of chemokine receptors, such as CXCR1 and CXCR2, was confirmed by quantitative PCR (Fig. 2B). Furthermore, high surface expression of CXCR2 in TIARP^−/−^ neutrophils was also detected by FACS (Fig. 2C). In addition, up-regulation of CCR1, CCL3 and CXCL12 was also confirmed by quantitative PCR (Supplementary Figure 1A). Considered together, the quantitative PCR data sets were largely concordant with the results of microarray analysis.

Among the factors involved in the migration of neutrophils out of the vasculature and into tissues, the β2 integrin LFA-1 (CD11a/CD18) is important in the tight adhesion of neutrophils to the vascular endothelium that precedes extravasation into tissues^25^. LFA-1 was strongly expressed in TIARP^−/−^ neutrophils, compared with WT neutrophils (Supplementary Figure 1B). These findings suggest possible involvement of CXCR1/2 expression and LFA-1-mediated adhesion of neutrophils in the development of arthritis in TIARP^−/−^ mice.

### Enhanced migration capacity of TIARP^−/−^ neutrophils via CXCL2

We further explored whether TIARP^−/−^ neutrophils have potentially strong migration capacity into the site of inflammation. The neutrophil chemoattractant ligand for CXCR1 and CXCR2 is CXCL2 (also known as MIP-2; a chemokine related to human IL-8; CXCL8). Transmigration assays showed that CXCL2 increased the chemotactic response of TIARP^−/−^ neutrophils, compared to WT neutrophils (Fig. 2D). Previous studies investigated the optimal dose of intraperitoneally injected CXCL2 that would cause peritoneal neutrophil migration^26^. To examine further the role of CXCL2 in neutrophil migration *in vivo*, 0.5 μg of CXCL2 in 200 μl PBS alone was injected intraperitoneally and peritoneal neutrophils and macrophages were counted 4 hr later. The number of peritoneal exudate cells (PECs) increased after the injection of CXCL2 (Fig. 2E). Moreover, CXCL2 significantly increased peritoneal neutrophil and macrophage counts. On the other hand, the number of CD3^+^ T cells was not different between WT and TIARP^−/−^ mice (Fig. 2F). Together, these findings suggest that TIARP^−/−^ neutrophils (also to some extent macrophages) that constitutively express CXCR1 and CXCR2 seem to enhance their recruitment into CXCL2-rich site.

Next, we assessed the effects of TIARP on other functions, such as the production of inflammatory cytokines and reactive oxygen species (ROS) by neutrophils. Neutrophils from splenocytes were collected and incubated for 5 hours with mPAP-IC. The production of IL-17, TNFα, and IL-6 concentrations in the culture supernatants, as measured by ELISA was comparable between WT and TIARP^−/−^ mice (Supplementary Figure 2A).

Since the oxidase function of NADPH and production of ROS by neutrophils is implicated in the pathology of RA^27^, we examined next the role of TIARP in ROS production. The results showed no increase in neutrophils in TIARP^−/−^ mice (Supplementary Figure 2B). K/BxN serum-transferred arthritis is also mediated by cell surface Fc-receptors^21^. Mouse neutrophils express both stimulatory (FcγRI, FcγRII, and FcγRIII) and inhibitory (FcγRIIb) Fc receptors. FcγRIII is important for the binding of immune complexes, associated mobilization of calcium, and downstream effects such as oxidative burst^23^. Next, we analyzed the surface expression of FcγR on neutrophils to determine its role in this system. The expression levels of FcγRI, II and III were comparable between WT and TIARP^−/−^ neutrophils (Supplementary Figure 2C). Together, these observations suggest that TIARP deficiency neither enhances the production of these cytokines or ROS, nor the expression of FcγR.

### Pathogenic role of FLS in progression of serum-transferred arthritis in TIARP^−/−^ mice

**Figure 3:**
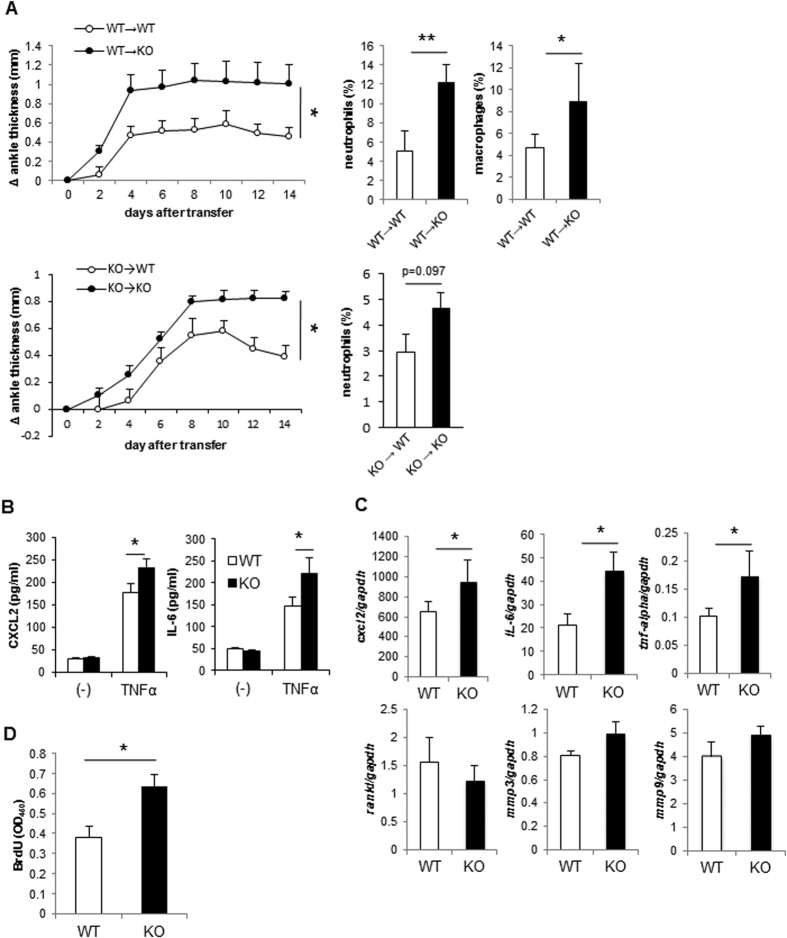
High production of CXCL2 in TIARP^−/−^ TNFα-stimulated FLS. (**A**) Lethally irradiated WT and TIARP^−/−^ mice (CD45.2^+^) received WT (CD45.1^+^) bone marrow (BM), or bone marrow (BM) from TIARP^−/−^ mice into WT or TIARP^−/−^ mice. Serum-transferred arthritis was induced after 6 weeks of irradiation. (**B,C**) FLS were isolated from the ankle joints and stimulated with 100 ng/ml TNFα for 24 hr. Chemokine and cytokine expression levels were analyzed by ELISA (**D**) and qPCR (**E**). (**F**) Proliferative response of FLS after the addition of 100 ng/ml TNFα was assessed by BrdU incorporation. Data are mean ± SEM of two experiments. *P < 0.05, **P < 0.01.

We reported previously the expression of TIARP in the synovial of arthritic joints in both mice and humans^13^. However, there is no information on the role of non-myeloid cells (such as fibroblast-like synoviocytes (FLS)) in serum-transferred arthritis in TIARP^−/−^ mice, and whether cytokine or chemokine production by these cells play a role in arthritis. To assess the role of non-myeloid cells of TIARP^−/−^ in the generation of serum-transferred arthritis, bone marrow (BM) from WT (CD45.1) mice were adoptively transferred into WT or TIARP^−/−^ mice (CD45.2). TIARP^−/−^ mice that received WT BM developed more severe arthritis than WT mice that received WT BM (Fig. 3A, upper column), suggesting that these cells play an important role in the pathogenesis of arthritis in TIARP^−/−^ mice. The percentage of neutrophils and macrophages were increased in TIARP^−/−^ mice that received WT BM. In addition, we examined another set of experiments with bone marrow (BM) from TIARP^−/−^ mice into WT or TIARP^−/−^ mice. TIARP^−/−^ mice adoptively transferred TIARP^−/−^ mice also developed more severe arthritis than WT mice that received TIARP^−/−^ mice, however, the numbers of neutrophils were not statistically different (Fig. 3A, lower column). These experiments suggest that important role of TIARP^−/−^non hematopoietic cells in arthritis and cell migration to the joint in this system, but hematopoietic cells in TIARP^−/−^ mice also enhance arthritis.

Previous studies indicated that IL-8 (a counterpart of mouse CXCL2) and IL-6 are induced by TNFα in FLS from RA patients^26^,^28^,^29^, and STEAP4 (human ortholog of TIARP) knock-down enhance the production of IL-8 and IL-6 by TNFα stimulation^14^. Next, we explored the effects of TIARP on CXCL2 and IL-6 production by FLS. As anticipated, the production of CXCL2 and IL-6 by TIARP^−/−^ FLS treated with TNFα was significantly higher than WT FLS (Fig. 3B). Furthermore, the expression levels of CXCL2, IL-6 and TNFα in FLS were significantly higher in TIARP^−/−^ FLS than WT (Fig. 3C). In contrast, the expression of genes related to bone destruction (such as RANKL, MMP3 and MMP9) was not different between WT and TIARP^−/−^ FLS (Fig. 3C). In addition, the proliferative response to TNFα was higher in TIARP^−/−^ FLS than WT FLS (Fig. 3D). These results suggest that TIARP deficiency on FLS enhances the production of CXCL2 and IL-6, as well as cell proliferation.

We also compared the chemotactic activity of supernatants of FLS with or without TNFα, and detected enhanced cell migration in neutrophils co-cultured with supernatants from FLS incubated with TNFα, but not unstimulated FLS (Supplementary Figure 3). Moreover, we also examined whether TNFα alters the chemokine receptor expression on TIARP^−/−^ neutrophils. However, CXCR1 and CXCR2 expression levels were not upregulated in both WT and TIARP^−/−^ neutrophils after stimulation with TNFα (Supplementary Figure 4). These data suggest that TNFα requires the production of CXCL2 by FLS without affecting the expression of chemokine receptors on neutrophils.

### Enhanced chemotactic activity of TIARP^−/−^ neutrophils, and its abrogation by CXCL2-neutralization

**Figure 4:**
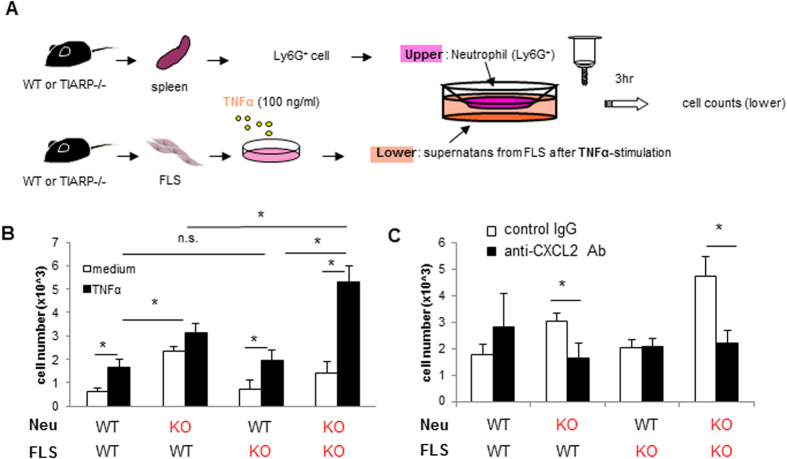
Enhanced chemotactic activity in TIARP^−/−^ neutrophils and its blockade by CXCL2-neutralization. (A) Experimental scheme. Criss-cross experiment was performed using neutrophils and FLS from WT and TIARP^−/−^ mice. The former cells were added to the upper chamber of the transwell apparatus, while supernatants from FLS stimulated with or without TNFα were added to the lower chamber. Three hours later, the number of migrated cells were counted (**B**). The same experiment was performed after adding anti-CXCL2 Ab to the lower chamber (**C**). Values are mean ± SEM. *P < 0.05.

To confirm the effects of TIARP on migration of neutrophils into the joint synovium, we performed criss-cross experiments using neutrophils as well as FLS from WT and TIARP^−/−^ mice (Fig. 4A). Neutrophils derived from splenocytes of WT and TIARP^−/−^ mice were seeded in the upper well, while twice-diluted supernatants from WT and TIARP^−/−^ FLS were placed in the lower wells, in the absence or presence of TNFα. For activating FLS, we also used IL-6 and LPS. However, IL-6 could not induce CXCL2 (Supplementary Figure 5) from FLS even from TIARP^−/−^ *in vitro*, and neutrophil migration was not induced by TIARP^−/−^ FLS with IL-6 stimulation indeed (Supplementary Figure 6). In contrast, TNFα clearly induce CXCL2 as well as IL-6 from FLS *in vitro* (Fig. 3B), and neutrophil migration (especially TIARP^−/−^) was clearly dependent on TNFα-stimulation. To confirm the relevance of IL-6 in this vitro CXCL2 production system, we tried several conditions such as TNF + IL-6 and TNF + anti-IL-6R (this experiments prove the autocrine response of IL-6 *in vitro*). As a result, IL-6 signal did not relate to CXCL2 production by TNFα stimulation in FLS (supplementary Figure 5B,C). Thus, we used TNFα in Fig. 4 experiment *in vitro*. The number of TIARP^−/−^ neutrophils that migrated to the lower chamber was significantly higher than WT neutrophils, irrespective of the source of co-cultured FLS (Fig. 4B). Furthermore, supernatants from TIARP^−/−^ FLS markedly augmented chemotaxis of TIARP^−/−^ neutrophils (Fig. 4B). To further confirm the role of CXCL2 in neutrophil migration, we performed chemotaxis assay after the addition of anti-CXCL2 antibodies or control IgG to the lower chamber. CXCL2-neutralizing antibodies, but not the control antibody, completely abrogated the enhanced recruitment of TIARP^−/−^ neutrophils (Fig. 4C). These results suggest that neutrophil migration to the site of inflammation in TIARP^−/−^ mice is mediated through overproduction of CXCL2.

### Role of IL-6 in neutrophil intra-articular migration in serum-transferred arthritis in TIARP^−/−^ mice

**Figure 5:**
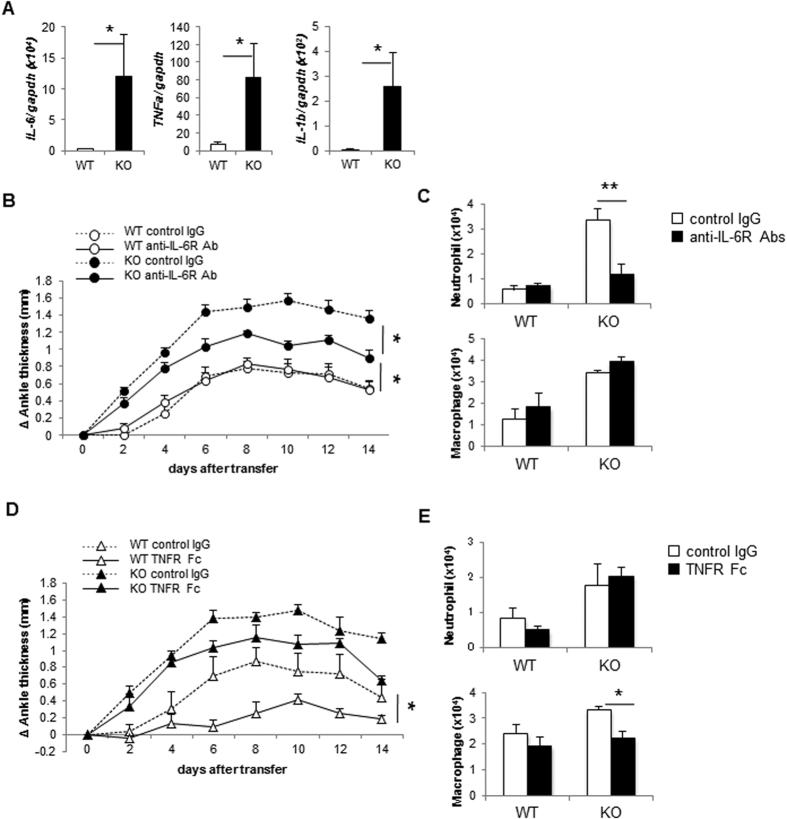
Severity of arthritis in TIARP^−/−^ mice correlated with inflammatory cytokine production. (**A**) Expression of TNFα, IL-6 and IL-1β mRNAs in the joints on day 14 analyzed by qPCR. Mice were administrated anti-IL-6R Ab (**B**), or TNFR-Fc (**D**). The dose of Abs was 2 and 1 mg/injection every 2 days, respectively. The numbers of migrated neutrophils and macrophages in the joint were counted on day 14 after treatment with anti-IL-6R Ab (**C**), TNFR-Fc (**E**). Data are mean ± SEM of two experiments. *P < 0.05, **P < 0.01.

Previous studies on K/BxN serum-transferred arthritis examined the roles of IL-1 and TNFα, but not IL-6, in disease development^17^,^24^. In comparison, experimental evidence indicate the involvement of IL-6 in CIA-TIARP^−/−^ mice^13^. To determine the role of IL-6, we analyzed the expression of inflammatory cytokines in the joints of K/BxN serum-transferred TIARP^−/−^ arthritic mice. The expression levels of TNFα, IL-6 and IL-1β were significantly higher in TIARP^−/−^ than WT mice (Fig. 5A).

To further explore the role of inflammatory cytokines in the generation of serum-transferred arthritis, blocking Abs for these cytokines were administrated in mice after the induction of arthritis. Surprisingly, administration of IL-6R Ab, but not control Ab, resulted in significantly attenuated the development of serum-transferred arthritis in TIARP^−/−^ mice. In comparison, no significant changes were observed in WT mice between anti-IL-6R and control Ab (Fig. 5B). Moreover, IL-6R administration clearly diminished intra-articular neutrophil recruitment, but not macrophages, in TIARP^−/−^ mice compared with WT mice (Fig. 5C). Furthermore, administration of TNFR-Fc failed to inhibit the development of arthritis in serum-transferred TIARP^−/−^ mice (Fig. 5D). In contrast, TNFR-Fc suppressed serum-transferred arthritis in WT mice. The number of neutrophils in the joint was comparable between TIARP^−/−^ mice injected with control IgG and TNFR-Fc. In comparison, the number of macrophages tended to decrease after TNFR-Fc treatment, compared with control IgG treatment (Fig. 5E). These results suggest that IL-6 signaling, but not TNFα, is critical for the recruitment of neutrophils into the arthritic joint in serum-transferred arthritis in TIARP^−/−^ mice.

## Discussion

In this study, we identified exacerbation of arthritis in K/BxN serum-transferred TIARP^−/−^ mice, and that such feature was mainly due to marked infiltration of neutrophils. Microarray analysis of TIARP^−/−^ neutrophils showed strong up-regulation of chemokine receptors, especially CXCR2 expression. The heightened intra-articular inflammatory response was mediated by up-regulation of CXCR2 in neutrophils, as well as production of CXCL2 by FLS both *in vitro* and *in vivo*. Our results also showed enhanced migration capacity of TIARP^−/−^ neutrophils, which was mediated mainly through CXCL2, and that IL-6 signaling is critical for the recruitment of neutrophils into the arthritic joints after serum-transferred arthritis in TIARP^−/−^ mice.

TIARP^−/−^ neutrophils constitutively expressed CXCR2 and had highly migration capacity. In a previously described mouse model that lacked CXCR2 but retained normal C5a and LTB4 receptors, intraperitoneal injection of thioglycollate resulted in 80% reduction in peritoneal neutrophil migration compared with control mice^30^. CXCR2 signaling pathway is known to induce changes in LFA-1 conformation that contribute to neutrophil adhesion and recruitment^31^. Indeed, our study showed enhanced LFA-1 expression in TIARP^−/−^ neutrophils. Chemokines mediate their biologic effects via CXCR2, which appear to play major roles in neutrophil recruitment during arthritis^32^. Neutrophil entry into joint tissue is the first step in this process, as demonstrated in various animal models of arthritis^33^,^34^,^35^. Thus, neutrophil chemoattractants are attractive targets for not only the prevention of inflammatory diseases but also the development of therapies that can alleviate the symptoms of chronic inflammation. TIARP is a potentially suitable candidate for new treatment of chronic inflammation via suppression of neutrophil migration.

Wipke *et al*.^36^ showed that mouse peroxidase-anti-peroxidase (mPAP) immune complex (IC) activates neutrophils through Fcγ receptor to get autoantibodies into joints in K/BxN serum transferred model. IC promotes the production of certain cytokines, such as IL-17 and TNFα, from neutrophils. These complexes of immunoglobulins engage FcγR on the surface of neutrophils, triggering degranulation and production of ROS into the synovial fluid^27^. Several mouse studies have indicated that neutrophils are the source of IL-17 (e.g. LPS-induced lung inflammation^37^,^38^, acute kidney ischemia-reperfusion injury^39^, systemic histoplasmosis^40^, and early-stage arthritis^36^). However, our results showed that the IL-17-producing neutrophils (including other inflammatory cytokines) and ROS-activated neutrophils were not different between WT and TIARP^−/−^ mice. Moreover, the expression of Fcγ receptors on neutrophils was also comparable. Our previous study showed that TIARP deficiency leads to enhancement of NF-κB signaling and IL-6-induced STAT3 phosphorylation, whereas pErk1/2 production did not induce such changes^13^. On the other hand, after stimulation by immune complexes, the signaling pathways start with tyrosine phosphorylation of the ITAMs in the receptor-associated adaptor molecules by kinases of the SRC family. In addition to the calcium-dependent pathways, the RAS-RAF-MAPK-pathway is important for cell activation following FcγR crosslinking^41^.

Our results showed that non-myeloid cells, especially FLS, play an essential role in the development of arthritis. FLS contribute to the inflammatory microenvironment through the production of pro-inflammatory factors, activation or recruitment of other immunocytes, including neutrophils. Our results showed upregulation of CXCL2 in TIARP^−/−^ TNFα-stimulated FLS. CXCL2, a potent neutrophil chemoattractant, plays a pivotal role in the recruitment and activation of neutrophils and is considered the most important inflammatory chemokine associated with arthritis^42^,^43^,^44^. Other studies showed positive correlation between human IL-8 and the number of neutrophils in the synovial fluid of RA patients^45^,^46^. Furthermore, CXCL2 is essential for inflammation mediated by neutrophils^47^,^48^. In our vitro criss-cross experiment, we suppose that the magnitude of neutrophil trafficking was mainly dependent on CXCR2 in TIARP^−/−^ neutrophils, because blockade by anti-CXCL2 did not reflect neutrophil migration in TIARP^−/−^ FLS plus WT neutrophil experiment (Fig. 4C). In addition, CXCL2 produced by TIARP^−/−^ FLS *in vitro* is tiny amount (0.2 ng/ml, in Fig. 3B), and TIARP^−/−^ neutrophil could migrate well with 10 ng/ml (even if 0 ng/ml) concentration of CXCL2 (Fig. 2D).

How does TIARP inhibit CXCL2 production in FLS? High CXCL2 (also known as IL-8) expression has been reported in patients with RA, in whom neutrophils were the predominant cells in the joints^23^,^49^. Based on previous studies showing TNFα-induced CXCL2 production in RA FLS^26^,^50^, we evaluated here the role of TNFα in enhancing CXCL2 production in TIARP^−/−^ FLS. Activation of MAPK and NF-kB pathways contributes to CXCL2 expression^49^,^51^. Furthermore, TIARP^−/−^ macrophages enhanced NF-kB signaling and increased IL-6 induced STAT3 phosphorylation^13^. We detected enhanced cell migration in neutrophils co-cultured with supernatants from FLS incubated with TNFα, but not unstimulated FLS. It is possible that other cytokines also mediate the production of CXCL2 by FLS. Considering that promotion of neutrophil migration into the site of inflammation is critical for strengthening the cross-talk between neutrophils and FLS, identification of new inhibitors that can suppress the production of CXCL2 could be helpful in the treatment of RA.

The effector phase of arthritis involves several molecular mediators including TNF and IL-1, but not IL-6^17^. However, the present results have demonstrated that blockade of IL-6R seems to have a therapeutic effect on serum-transferred arthritis with clear protection of neutrophil recruitment in joints of TIARP^−/−^ mice. In contrast, neutralization for TNFR had only partial effect on serum-transferred arthritis. In addition, our group has also reported that treatment with anti-IL-6R mAb, but not with TNFR-Fc, prevents the development of CIA^13^. IL-6 is a critical cytokine known to regulate Th17 development. Th17 is implicated as the driving force of autoimmune inflammation in several animal models such as CIA^52^, adjuvant-induced arthritis (AIA)^53^, and glucose-6-phosphate isomerase (GPI)-induced arthritis (GIA)^54^. However, serum-transferred arthritis is a neutrophil-dependent and T cell-independent model of RA^19^, suggesting that the effect of anti-IL-6R Abs in the development of serum-transferred arthritis in TIARP^−/−^ mice might be distinct from T helper cell differentiation.

In conclusion, we have demonstrated in the present study that TIARP can suppress the pathogenesis of neutrophils-mediated arthritis. Specifically, the results showed that infiltrating neutrophils and IL-6 form cross-talk, and that TIARP suppressed both CXCL2 production by FLS and FLS proliferation under inflammatory environment. These results suggest the importance of TIARP through the induction of CXCR2/ CXCL2 and IL-6 production. Our study provides new insights into the molecular mechanism of how TIARP controls tissue inflammation, such as RA.

## Additional Information

**How to cite this article**: Inoue, A. *et al*. TIARP attenuates autoantibody-mediated arthritis via the suppression of neutrophil migration by reducing CXCL2/CXCR2 and IL-6 expression. *Sci. Rep.* **6**, 38684; doi: 10.1038/srep38684 (2016).

**Publisher's note:** Springer Nature remains neutral with regard to jurisdictional claims in published maps and institutional affiliations.

## Supplementary Material

Supplementary Information

## Figures and Tables

**Table 1:** GO enrichment and functional annotation analysis of up-regulated genes in TIARP^−/−^ neutrophils.

No.	Term	P-Value	Benjamini
1	chemotaxis	0.000017	0.027
2	taxis	0.000017	0.027
3	immune response	0.00005	0.041
4	gas transport	0.00011	0.06
5	response to wounding	0.00099	0.33
6	oxygen transport	0.0013	0.35
7	erythrocyte differentiation	0.0031	0.57
8	cell adhesion	0.0034	0.55
9	biological adhesion	0.0035	0.51
10	myeloid cell differentiation	0.0039	0.51
11	erythrocyte homeostasis	0.0041	0.49
12	locomotory behavior	0.0044	0.48
13	myeloid leukocyte activation	0.0058	0.55
14	defense response	0.0061	0.54
15	hemopoiesis	0.0065	0.53
16	inflammatory response	0.0076	0.57
17	homeostasis of number of cells	0.0093	0.62
18	positive regulation of cell motion	0.01	0.63
19	protein targeting to membrane	0.01	0.61
20	triglyceride biosynthetic process	0.011	0.62
21	lymphocyte costimulation	0.011	0.62
22	T cell costimulation	0.011	0.62
23	glycerol metabolic process	0.012	0.62
24	polyol metabolic process	0.012	0.61
25	cytokine production	0.012	0.61
26	alditol metabolic process	0.015	0.67
27	hemopoietic or lymphoid organ development	0.015	0.66
28	secretion by cell	0.016	0.67
29	macrophage activation	0.017	0.69
30	secretion	0.018	0.68
31	leukocyte chemotaxis	0.018	0.67
32	cell chemotaxis	0.018	0.67
33	regulation of response to external stimulus	0.021	0.71
34	immune system development	0.021	0.7
35	biogenic amine biosynthetic process	0.022	0.7
36	behavior	0.023	0.7
37	positive regulation of secretion	0.024	0.72
38	regulation of cell motion	0.024	0.71
39	innate immune response	0.024	0.71
40	myeloid cell activation during immune response	0.025	0.7
41	neutral lipid biosynthetic process	0.025	0.7
42	acylglycerol biosynthetic process	0.025	0.7
43	leukocyte activation during immune response	0.026	0.71
44	cell activation during immune response	0.026	0.71
45	regulation of locomotion	0.027	0.72
46	glycerol ether biosynthetic process	0.029	0.73
47	glycerolipid biosynthetic process	0.032	0.76
48	positive regulation of Ras protein signal transduction	0.033	0.76
49	positive regulation of small GTPase mediated signal transduction	0.038	0.8
50	leukocyte activation	0.04	0.81
51	positive regulation of cell migration	0.041	0.81
52	negative regulation of cell communication	0.041	0.8
53	regulation of cell migration	0.043	0.81
54	response to lipopolysaccharide	0.044	0.81
