# Supplementary material for: TIARP attenuates autoantibody-mediated arthritis via the suppression of neutrophil migration by reducing CXCL2/CXCR2 and IL-6 expression
Source: Sci Rep. 2016 Dec 20;6:38684. doi: 10.1038/srep38684 (PMC5171802; doi:10.1038/srep38684)

**SUPPLEMENTARY INFORMATION**

**TIARP attenuates autoantibody-mediated arthritis via the suppression of neutrophil migration by reducing CXCL2/CXCR2 and IL-6 expression**

Asuka Inoue^1^, Isao Matsumoto^1^, Yuki Tanaka^1^, Naoto Umeda^1^, Chinatsu Takai^1^, Hoshimi Kawaguchi^1^, Hiroshi Ebe^1^, Hiroto Yoshida^2^, Yoshihiro Matsumoto^2^, Seiji Segawa^1^, Satoru Takahashi^3^, Takayuki Sumida^1^

**Supplementary Figure 1.** Validation of the gene expression patterns of DNA microarray results.

(A) Gene expression levels of CCR1, CCL3 and CXCL12 validated by quantitative PCR, using independently collected neutrophils. (B) The expression level of LFA-1 (itgal/ itgb2) was analyzed by qPCR. Results were reproducible in three independent experiments. Data are mean±SEM. *P<0.05,

**Supplementary Figure 2.** No difference in cytokine and ROS production and FcγR expression between TIARP^-/-^ and WT neutrophils.

(A) Neutrophils collected from splenocytes were cultured for 5 hours by incubation with mPAP (murine peroxidase (HRP) –anti-peroxidase immune complex (IC)). The concentrations of IL-17, TNFα and IL-6 were measured by ELISA. (B) ROS activity (H_2_O_2_) was measured by stimulation of zymosan. Results were reproducible in three independent experiments. (C) Expression of FcγRIII, II, and I analyzed by flow cytometry. Results were reproducible in three independent experiments. Data are mean±SEM.

**Supplementary Figure 3.** Role of TNFα in TIARP-related chemotactic ability.

We performed transmigration assays using neutrophil and FLS from WT and TIARP^-/-^ mice. Neutrophils were added to the upper chamber of the transwell apparatus, while supernatants from FLS stimulated with or without TNFα was added to the lower chamber. Three hours later, the number of migrated cells were counted. Data are mean±SEM. *P<0.05,

**Supplementary Figure 4.** TNFα stimulation does not induce chemokine receptor expression on neutrophils.

Neutrophils isolated from WT and TIARP^-/-^ mice were pre-incubated with TNFα for 3 hr. The expression levels of CXCR1 and CXCR2 were analyzed by qPCR. Data are mean±SEM of two experiments.

**Supplementary figure 5**

CXCL2 expression of FLS harvested from WT and TIARP^-/-^ mice. (A) FLS were cultured in the presence of 100 ng/ml LPS, 100ng/ml TNFα or 10ng/ml IL-6 and prepared for real-time PCR analysis. (B)(C) FLS were isolated from the ankle joints and stimulated with 100 ng/ml TNFα and/or 10ng/ml IL-6 and/or 1μg anti-IL-6R for 24 hr. CXCL2 expression levels were analyzed by ELISA. Data are mean ± SEM of two independent experiments (n=5 per experiment). *: *P*<0.05.

**Supplementary figure 6**

Chemotaxis experiment was performed using neutrophils and FLS from WT mice. The former cells were added to the upper chamber of the transwell apparatus, while supernatants from FLS stimulated with LPS, TNFα or IL-6 were added to the lower chamber. Three hours later, the numbers of migrated cells were counted.


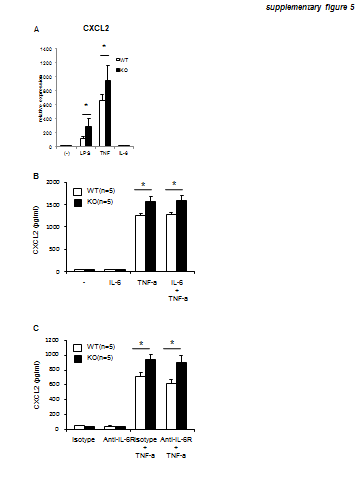

Supplement: Supplementary Information [file srep38684-s1.doc]
